# The Contribution of Inter-ply Friction to Deformation Modes During Double Diaphragm Forming of Thick Non-crimp Fabric Preforms

**DOI:** 10.1007/s10443-026-10495-w

**Published:** 2026-07-02

**Authors:** Guy Donald Lawrence, Shuai Chen, Lee Thomas Harper

**Affiliations:** 1 Composites Research Group, Faculty of Engineering, University of Nottingham, Nottingham, NG7 2RD UK; 2 International Center for Machinery, School of Mechanical Engineering, Xi’an Jiaotong University, Xi’an, Shaanxi Province P.R. China

**Keywords:** Fabrics/Textiles, Forming, Friction, Defects

## Abstract

This study investigates the contribution of inter-ply friction to the dominant deformation mechanisms in multi-ply non-crimp fabric (NCF) layups during the forming of complex three-dimensional geometries. A modified cantilevered bending test was developed to assess the influence of increasing inter-ply friction on the non-linear bending behavior of multi-ply stacks. Furthermore, the effect of inter-ply friction on in-plane shear behavior was examined experimentally under vacuum pressure, simulating the conditions encountered during double diaphragm forming (DDF). The results demonstrate that both bending stiffness and shear resistance of the laminate stack increase with enhanced ply compaction, which generates greater inter-ply frictional forces. A DDF case study involving a 16-ply NCF preform highlights the impact of elevated bending stiffness and shear resistance on formability. Notably, increased frictional forces arising from variations in ply stacking sequences can adversely affect formability, as evidenced by the formation of pronounced out-of-plane wrinkles. This systematic investigation reveals that the final geometry of the demonstrator preform is governed more significantly by in-plane shear resistance than by out-of-plane bending behavior. These findings underscore the critical role of inter-ply friction in shaping the deformation response of multi-ply NCFs and offer insights for optimising preform design and processing strategies in advanced composite manufacturing.

## Introduction

Non-crimp fabrics (NCF) comprise multi‑directional fibre layers held together by stitches, rather than yarn interlacement, thereby avoiding crimp. These materials tend to have superior in-plane properties compared to woven fabrics [1]. The primary role of the meso-scale stitch is to aid handling of the fabric and to facilitate composite manufacturing, rather than to improve mechanical performance. The main incentive for using NCFs is therefore to reduce overall composite manufacturing costs, since thicker, heavier fabrics can be used over woven materials, enabling the number of individual plies to be minimised and fibre deposition rates to be maximised. The major downside to using NCFs is reduced formability [2], affecting the likelihood of producing complex components without defects like out-of-plane wrinkles, in-plane fibre waviness and fabric-tool bridging [3, 4]. Forming multiple plies with dissimilar ply orientations further increases complexity, with the introduction of additional frictional and through-thickness compaction deformation modes to consider.

Most composite forming studies in the literature focus on just one or two fabric plies. Early work by Prodromou et al. [5] established the relationship between shear angle and wrinkling in a single fabric layer, demonstrating that plies tend to buckle out-of-plane when the shear angle approaches the locking point. The shear-wrinkling relationship for individual fabric plies has continued to be of interest for many different materials [6–9], indicating that it is a critical mechanism when considering fabric formability. A mesoscale FE model was developed to reproduce the shear mechanisms for a single biaxial NCF ply [10], indicating the tensile contribution from the stitch yarn on the overall in-plane shear behaviour of the fabric. High-fidelity mesoscale material models [11–13] are valuable for modelling these effects, but they are generally too computationally expensive for multi-ply, full-scale forming simulations. Lower fidelity models are therefore required, but the deformation modes need to be thoroughly understood before the material behaviour can be homogenised to take advantage of lower-order elements.

Wrinkle development in multi-ply preforms strongly increases if the plies have different orientations [14, 15]. The coefficient of friction is fibre orientation dependent [16], as the relative angle between adjacent plies affects the nesting behaviour and therefore the degree of fibre-fibre contact [17]. Validated numerical simulations demonstrate that slippage between plies of different orientations generates local compressive forces in the fabric, resulting in wrinkles [18, 19]. The use of a blank holder can exacerbate the wrinkles further, as higher frictional forces result from the increased normal load. Several other studies have demonstrated how high inter-ply frictional forces within a multi-ply stack are undesirable and can affect the onset of wrinkling [5, 20, 21]. Part of the solution is to reduce the inter-ply coefficient of friction by lubricating [22] or reducing fibre nesting [17], enabling greater levels of inter-ply sliding to alleviate out-of-plane wrinkles and improve overall formability.

Alshahrani and Hoijati [23] have shown that the shear resistance of woven prepreg is pressure sensitive, due to the increase in intra-ply friction at the yarn crossover points as the fabric is compacted. A modified bias extension test was developed to study the influence of compaction force on the deformation limit and wrinkling onset point of the fabric when subjected to similar conditions to the diaphragm forming process. It was concluded that fabric compaction should be taken into consideration during future forming simulations, as the in-plane shear deformation of the fabric is severely reduced due to the normal pressure. It is not fully understood if the same phenomenon is true for NCFs, since the yarn fixation method is quite different and there is no fibre undulation, so the constraint of the rotation and sliding of the primary yarns is quite different. In addition, compacting the fabric changes the shape of the fibre bundles, increasing the fibre-to-fibre contact area and consequently the inter-ply frictional forces, which limits sliding during forming [17].

The bending stiffness of biaxial NCFs is nonlinear as ply curvature increases [24–29]. This phenomenon occurs due to fibre buckling and slippage of the yarns and must be accounted for when using macroscale continuum models if out-of-plane fabric wrinkling is to be captured effectively [22, 28]. Liang et al. [30] considered the bending stiffness of both single and multi-ply stacks of 3D woven angle interlock materials. The bending stiffness of the multi-ply stack was greater than the summation of the bending stiffness of the individual plies due to the frictional moment, which is a significant component of the total bending moment. Shen et al. [29] demonstrated that the increase in frictional force from through-thickness tufting yarns was the primary factor influencing the bending behavior of multilayered reinforcements. Similar behaviour was observed for the bending response of a multi-layered book-like system [31], demonstrating an upper and lower limit for the bending stiffness of the stack. The ply stack acts as a solid beam when the inter-ply friction is high enough to prevent slip (upper limit), for example due to the tackiness between prepreg plies [32], whereas each layer bends independently when the friction is zero (lower limit). It is necessary to take this friction contribution into account when simulating the bending behaviour of composite reinforcement, which can be achieved by homogenising the material behaviour through the thickness and adopting a global multilayer bending curve [30]. However, this is not practical for complex layups with varying numbers of plies at different orientations, as the bending properties strongly depend on the ply layup sequence [32], therefore a different calibrated material model would be required for each case. The influence of inter-ply friction on the out-of-plane bending deformation is therefore not well understood.

This paper aims to investigate interactions between inter- and/or intra-ply friction and other common NCF deformation modes, including bending and in-plane shear, which occur during the forming of multi-ply laminates. Existing coupon tests are modified to incorporate these interactions, including a modified cantilever bend test and bias extension test performed under vacuum consolidation conditions. The objective is to produce fabric deformation conditions that are more representative of those experienced during the Double Diapragm Forming (DDF) process [33–35], to provide more realistic material input data and better validate future simulation activities. A case study is presented to evaluate the effect of frictional forces on the formability of a representative preform geometry.

## Methodology

### NCF Material

A biaxial NCF material is used for the study, which consists of carbon fibres at ± 45° and a polyester pillar stitch in the 0° (roll) direction. The fabric is designated FCIM359 and was provided by Hexcel Reinforcements, Leicester, UK. Further details are presented in Table 1. An Assyst Bullmer XCUT automated ply cutter was used to cut all samples to ensure repeatability and minimise preparation time.

**Table 1:**
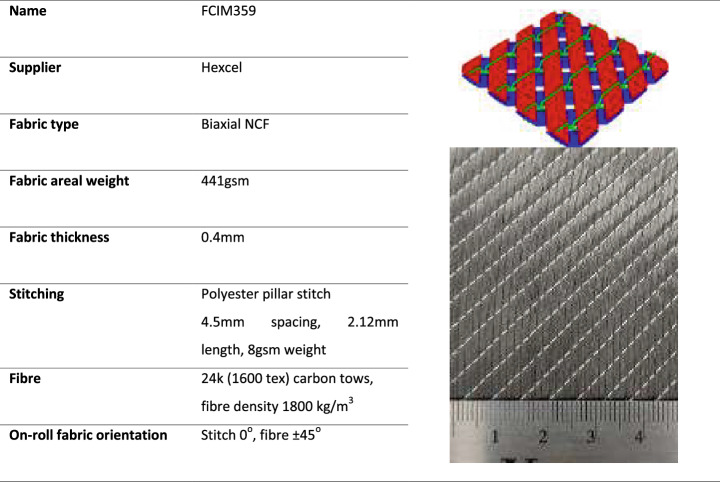
Material properties of the biaxial NCF used in this study. A schematic shows the primary yarn directions relative to the pillar stitch. The photo indicates the true yarn spacings

### Multi-ply Bend Test

Inter-ply friction influences the bending stiffness for multi-ply stacks, generating theoretical upper and lower bounds depending on the level of slip [36]. The upper limit causes the stack to effectively bend as a homogeneous beam, as slip between the layers is prevented by high levels of friction. The second moment of area for this case is as follows:

1 $$\:{I}_{U}=\frac{b{\left(nt\right)}^{3}}{12}$$

where *\:b* is the ply width, *\:n* is the number of plies in the stack and *\:t* is the thickness of each ply.

The lower limit occurs when there is no friction between layers and therefore there is no resistance to inter-ply slip. The total second moment of area in this case is as follows:

2 $$\:{I}_{L}=\frac{b{\left(t\right)}^{3}}{12}$$

**Fig. 1:**
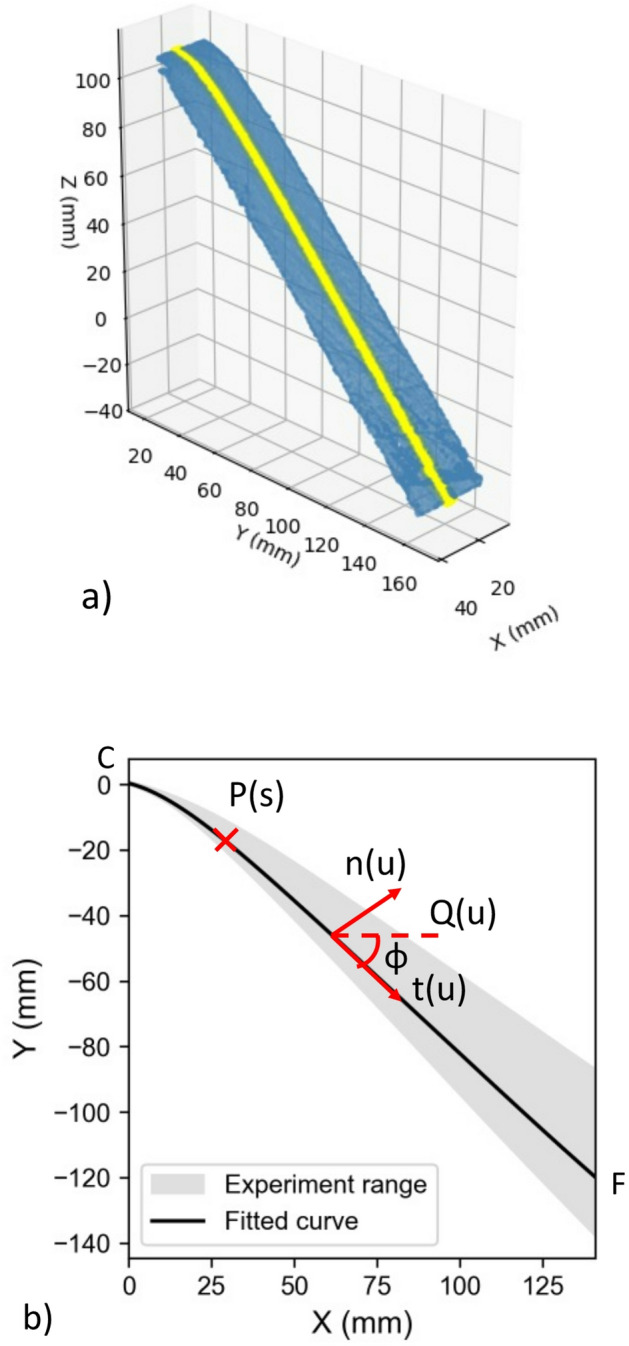
**a** Example scan of a bending sample for a single ply of FCIM359. **b** Free body diagram of the fitted curve from the 3D scan data for a [0°/90°] ply. Grey shaded region indicates the experimental range over 7 repeats

The bending stiffness of most real-life multi-layer beams will fall somewhere between these two limits, depending on the coefficient of friction between the layers. A modified cantilever bending protocol has been used to capture the curvature‑dependent stiffness of multiply NCFs, using laser-scanned side profiles of the fabric to extract moment–curvature relationships [24, 28, 30]. A Creaform Handyscan 3D Silver Series laser scanner was used to digitise the bending profile of the top ply (Fig. 1a). The resolution of the scanner was up to 0.1 mm. Specimens were cut to 250 mm × 30 mm and the overhang length was fixed at 200 mm to obtain a sufficiently wide range of curvature over each specimen. A white powder spray (Ambersil Flaw Developer 3) was applied to the material to minimise reflections from the surface of the carbon fibre during scanning. No difference in bending behaviour was observed when the spray was applied. Only the central 10% of data points were used to produce the bending profile, reducing the influence of any unwanted twist or rotation in the fabric. A deflection curve was obtained by fitting a 6th order polynomial to the data points for each sample, as per previous studies for the same NCF material [28]. A minimum of 3 repeats were conducted for each cantilever bend test configuration.

**Fig. 2:**
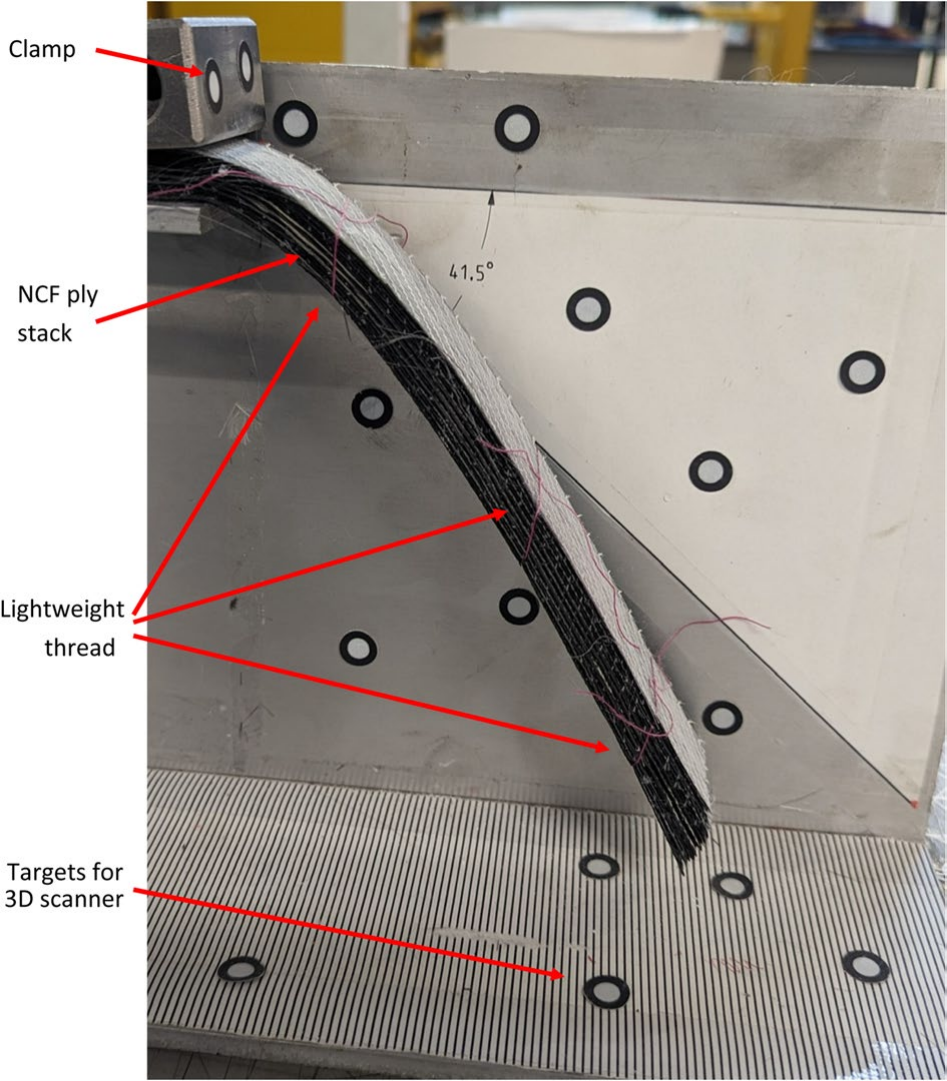
A multi-ply bending sample of FCIM359. Loops of lightweight thread are used to prevent deconsolidation of the stack. Reflective circular targets are used to align images from the laser scanner

Loops of lightweight cotton thread (25 Tex – 25 g per km) were added around the multi-ply specimens to prevent deconsolidation, without hindering inter-ply slip, as shown in Fig. 2. The thread loops were tied loosely so that no compaction force was applied normal to the surface of the samples. The mass of the thread was considered negligible compared to the NCF specimen, and therefore it was assumed that any additional mass does not directly affect the bending stiffness, other than by encouraging resistance to inter-ply slip due to increased friction. Through-thickness tufts were also investigated for the same purpose, however, the lack of crimp in the NCF material resulted in too much instability in the sample and therefore the tufts had little effect.

The non-linear bending behaviour of FCIM359 is described as a function of the specimen curvature and the bending moment [24, 28]. Equation (3) was used to determine the curvature $\:\kappa\:$ at any given point along the bending profile, where $\:{y}^{{\prime\:}}$ and $\:{y}^{{\prime\:}{\prime\:}}$ are the first and second derivatives of the deflection curve with respect to *\:X*, as described by the free body diagram in Fig. 1b.

3 $$\:\kappa\:=\:\frac{{y}^{{\prime\:}{\prime\:}}}{{\left(1+{y}^{{\prime\:}2}\right)}^{\frac{3}{2}}}$$

To calculate the bending moment of the sample, first the accurate bending length of the deflection curve $\:{L}_{B}$ was determined, as per Eq. (4), where $\:{X}_{F}$ refers to the *\:X* coordinate at the free end of the beam and $\:{X}_{C}$ refers to the constrained end. $\:{L}_{B}$ should be equivalent to the overhang length of the sample, which is 200 mm for this case.

4 $$\:{L}_{B}={\int\:}_{{X}_{C}}^{{X}_{F}}\sqrt{1+{y}^{{\prime\:}2}}dx$$

From the bending length, the curvilinear abscissa *\:s* along the profile can be calculated, as shown in Eq. (5). Point *\:P* (shown on Fig. 1b), defined by $\:s=s\left(P\right)$, is the point about which the bending moment $\:M\left(s\right)$ applied by the beam *\:PF* and $\:\kappa\:$ are determined.

5 $$\:s\left(P\right)=\:{\int\:}_{{X}_{C}}^{{X}_{P}}\sqrt{1+{y}^{{\prime\:}2}}dx$$

Finally, Eq. (6) is used to determine $\:M\left(s\right)$, where $\:\varphi\:$ is the angle of the curve tangent to the horizontal and *\:W* is the specimen weight per unit length.

6 $$\:M\left(s\right)=\:W{\int\:}_{s}^{{L}_{B}}\left(u-s\right)\mathrm{cos}\left(\varphi\:\right)\:du$$

The resulting curvature versus bending moment relationship can be described using Voce’s model as per Eq. (7), where $\:{R}_{0\:}$ and $\:{R}_{Inf}$are fitting constants and $\:{\kappa\:}_{lim}$ is the exponential saturation parameter.

7 $$\begin{array}{c}\:M\left(\kappa\:\right)=\:{(R}_{0\:}\cdot\:\:\kappa\:)+\:{R}_{Inf}(1-\mathrm{e}\mathrm{x}\mathrm{p}(1-\frac{\kappa\:}{{\kappa\:}_{lim}}))\end{array}$$

### Multi-ply Shear Test

A modified bias extension test was employed to characterise the in-plane shear behaviour of a multi-ply laminate under DDF-like normal compaction. The standard bias extension methodology is described extensively by Cao. et al. [37]. Specimens were cut to 290 mm × 115 mm. The effective test area was 230 mm × 115 mm, providing 30 mm at either end to be clamped within the jaws. This also ensured that a ratio of 2:1 was maintained for both length and width. Tests were conducted on an Instron 5581 fitted with a 5kN load cell, using a crosshead speed of 10 mm/min. Five repeat specimens were tested for each scenario.

A shear angle ($\:\gamma\:$) versus shear force ($\:{F}_{Sh}$) curve was obtained from each experiment to describe the shear behaviour of the fabric, calculated using Eqs. (8) and (9).

8 $$\:\gamma\:=90-2{\mathrm{cos}}^{-1}\left(\frac{(H-W)+\delta\:}{\sqrt{2}(H-W)}\right)$$

9 $$\begin{array}{c}F_{Sh}\left(\gamma\:\right)=\:\frac1{\left(2H-3W\right)\mathrm{cos}\left(\gamma\:\right)}\\\left(\left(\frac HW-1\right)F\left(\mathrm{cos}\left(\frac{\gamma\:}2\right)-\mathrm{sin}\left(\frac{\gamma\:}2\right)\right)-WF_{Sh}\left(\frac{\gamma\:}2\right)\mathrm{cos}\left(\frac{\gamma\:}2\right)\right)\end{array}$$

where *H* and *W* are the height and width of a fabric sample respectively, $\:\delta\:$ is the crosshead displacement, *F* is the crosshead load and $\:{F}_{Sh}$ is the normalised shear force.

**Fig. 3:**
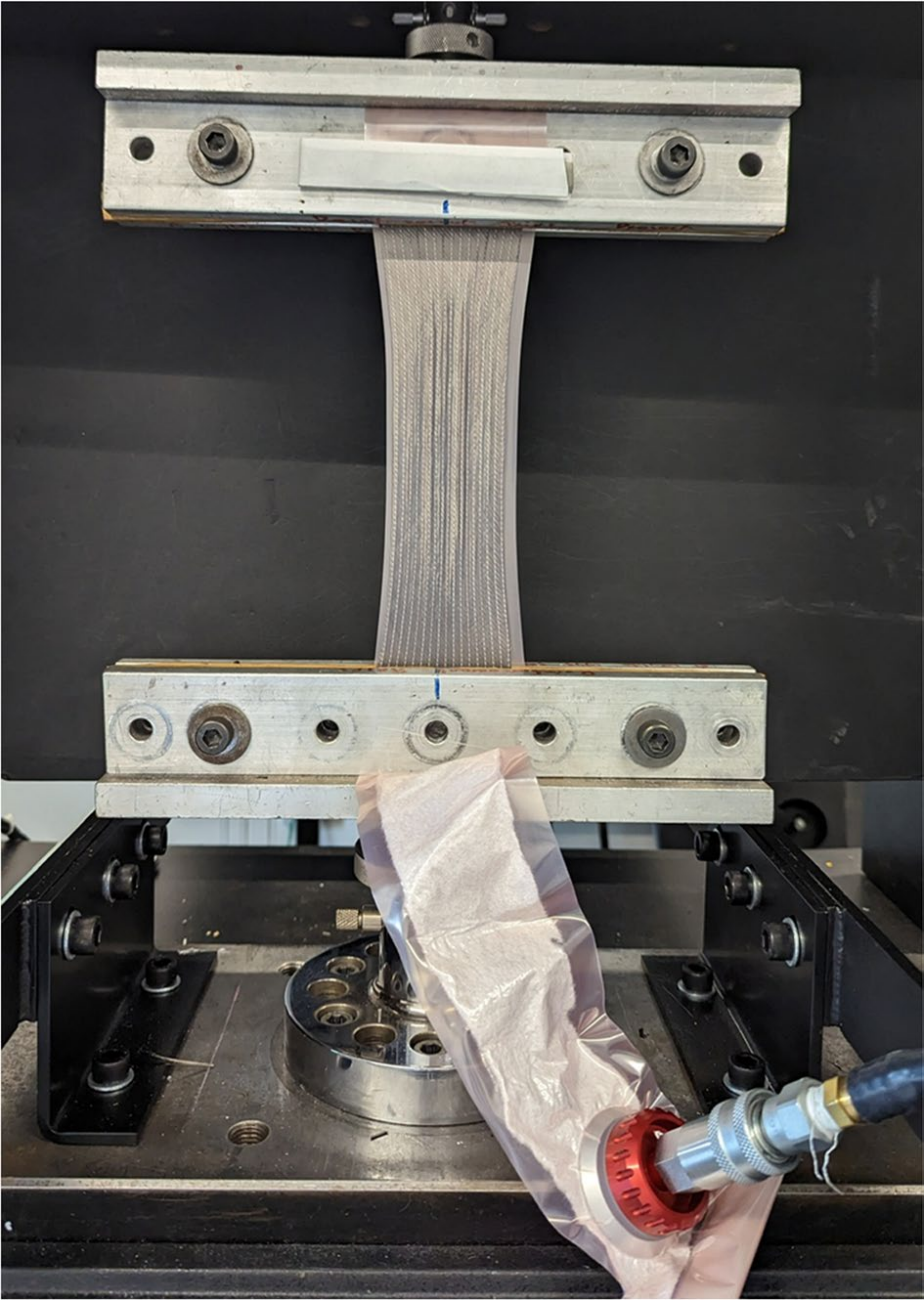
Bias extension test setup for a single ply of FCIM359. The ply is located within a vacuum bag sock, providing through-thickness compaction forces similar to the DDF process. The fabric is sheared by applying a tensile load to the entire assembly

The fabric samples for the bias extension test were placed inside a vacuum bag to simulate the compaction pressures experienced during DDF (Fig. 3). The force required to shear the sample can be described by Eq. (10) [23]:

10 $$\:{F}_{Sh}=F-{F}_{d}-{F}_{f}$$

which includes the additional load required to extend the vacuum bag ($\:{F}_{d}$) and the additional frictional force between the vacuum bag and the fabric sample ($\:{F}_{f}$) compared to the standard bias extension test.

**Fig. 4:**
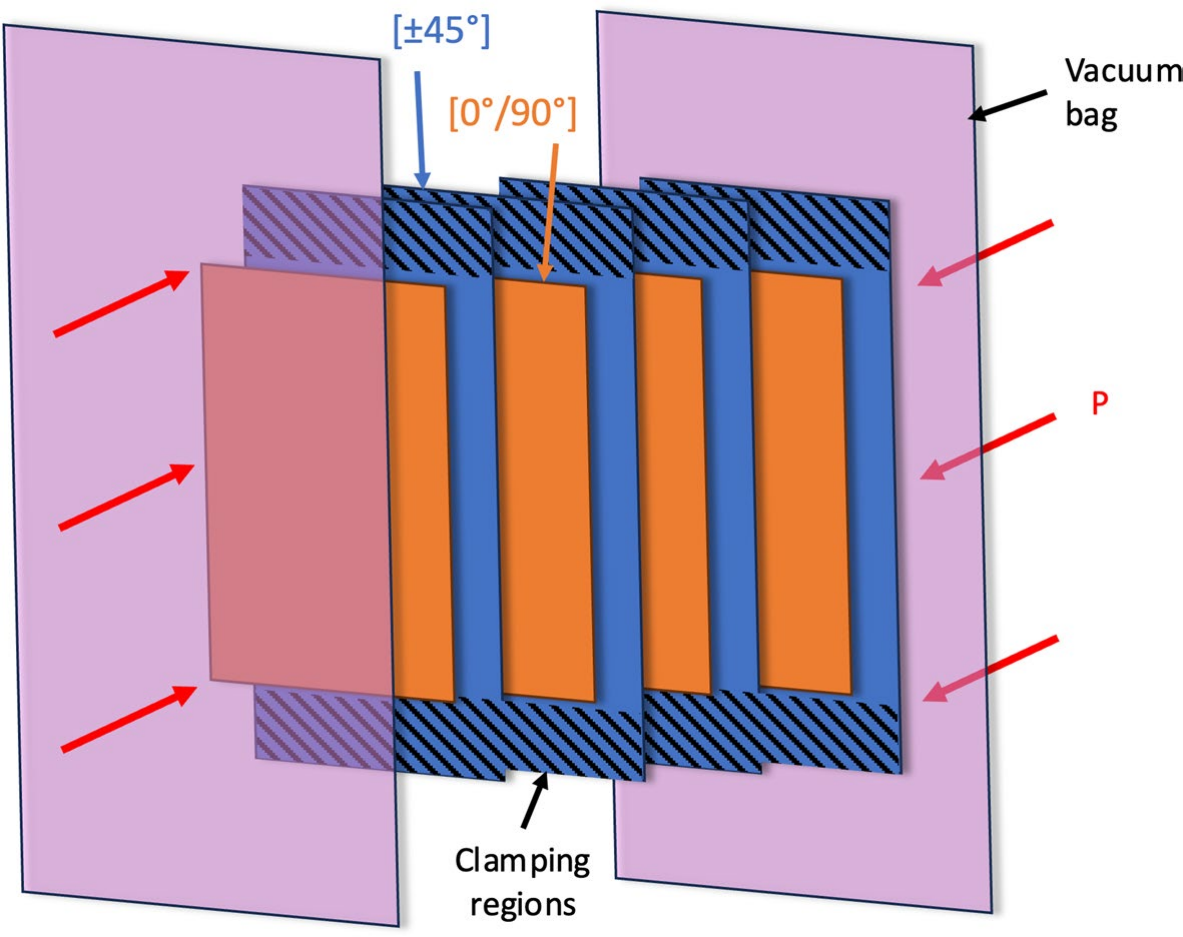
Schematic showing an example set up for a multi-ply bias extension test to assess the influence of inter-ply friction on in-plane shear behaviour. [± 45°] plies are clamped in the jaws of the testing machine. [0°/90°] plies are shorter and are not clamped in the jaws. The [0°/90°] plies are included to study the inter-ply frictional effects during in-plane shearing

Additional NCF plies were included for some bias extension tests to investigate the influence of inter-ply friction on the intra-ply shear behaviour. These fabric plies were orientated at [0°/90°] and were shorter (210 mm) than the original [± 45°] plies, as shown in Fig. 4. They were therefore unconstrained (i.e., not clamped at either end) to avoid the unwanted tensile contribution from the [0°/90°] fabric in the measured crosshead force. The layup sequence of the resulting fabric stack was varied to change the number of sliding interfaces between [0°/90°] and [± 45°] plies. For example, blocking all of the [0°/90°] plies together and all of the [± 45°] plies together created one sliding interface, whereas interleaving each [0°/90°] ply with a [± 45°] ply created the maximum 15 sliding interfaces.

### Double Diaphragm Forming

A laboratory-scale double diaphragm forming (DDF) machine was used to conduct experimental forming trials [38]. Stretchlon^®^ HT-350 was used for the diaphragm material, with dimensions of approximately 1.8 m x 1.5 m. Relevant material properties of Stretchlon^®^ HT-350 can be found in Table 2. The two diaphragms were clamped between three aluminium frames on the machine, which were then lowered into position over the male forming tool using four pneumatic cylinders.

**Fig. 5:**
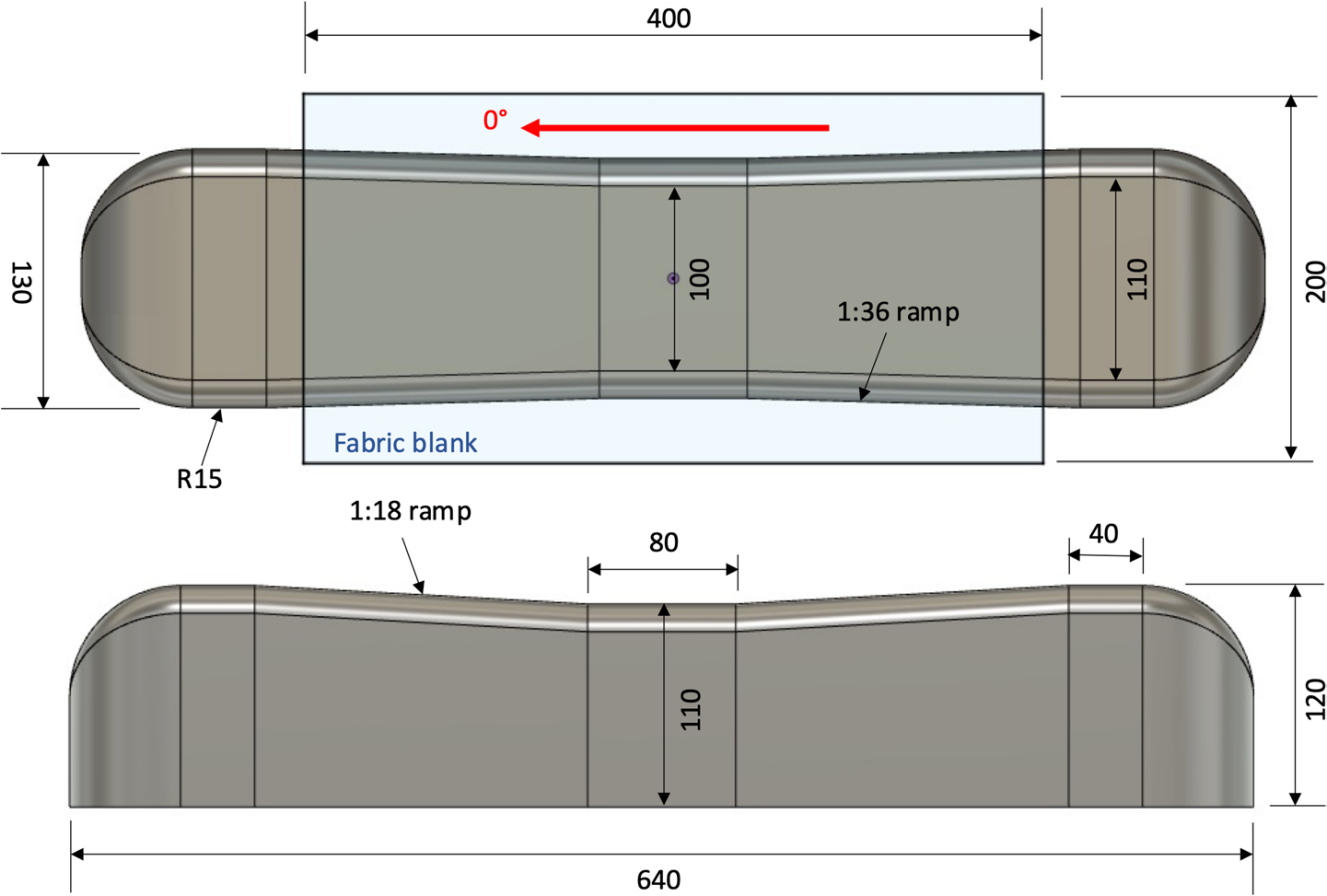
Geometry of male C-spar tool and fabric blank position used for experimental study (dimensions in mm). The principal fibre direction (0°) is indicated with an arrow

The male tool geometry used for the forming work is displayed in Fig. 5, which is similar to the geometry used by Johnson et al. [39]. The C-spar was selected as it is representative of a typical aerospace geometry that is difficult to form, including local sections with double curvature. A fabric blank size of 200 mm x 400 mm was used, which was aligned centrally with the spar geometry as shown in Fig. 5. The longitudinal axis of the spar was designated the 0° fibre direction.

**Table 2:** Material properties of the diaphragm material

Name	Stretchlon^®^ HT-350
Material type	Thermoplastic elastomer
Elongation at break	> 550%
Tensile strength	62 MPa
Maximum use temp.	162 °C

The Creaform laser scanner was used to digitise the geometry of the final DDF preforms. In-situ scanning within the DDF machine was conducted with the diaphragms in place (instead of stabilising the preform with a binder and removing it from the tool) to eliminate the effect of any uncontrolled preform relaxation and to improve the rate at which preforms could be tested. Additionally, the reduced reflectivity of the diaphragm material compared to the surface of the carbon fibre preform produced significantly higher quality scanning data.

**Fig. 6:**
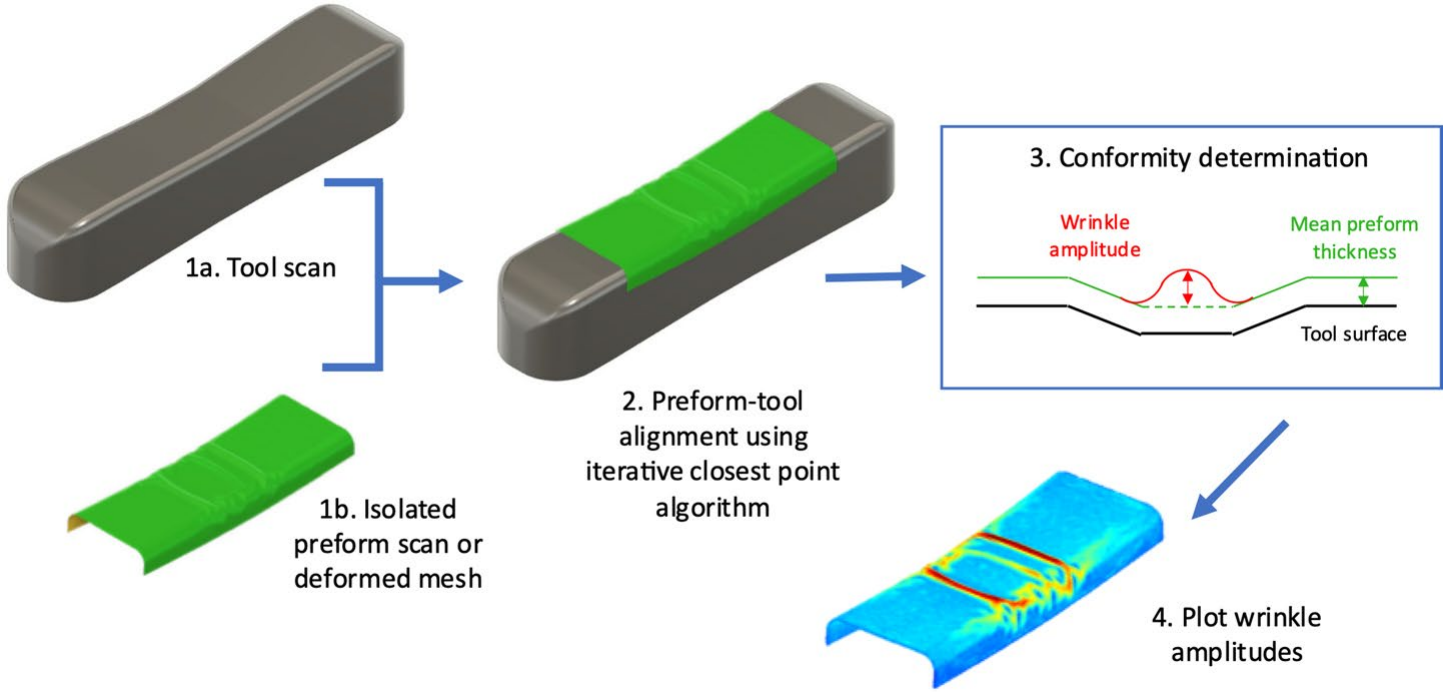
Workflow for in-situ preform wrinkle amplitude measurement. The tool geometry (1a) and preform scan (1b) are aligned using an iterative closest point algorithm (2). The normal distance between the two surfaces is used to calculate conformity and wrinkle amplitude (3) which is then represented as a colour map on the preform geometry (4)

The geometry was aligned to the principal axes using Creaform VX Elements scanning software, based on the centre of mass, producing a result such as the one shown in Fig. 6. The scan contained a portion of the tool surface that was used to assist with the alignment of the digital preform to the original geometry. An iterative ‘closest point’ algorithm in MATLAB was used to align the preform with its original position on the tool, identifying the normal distance between the outermost surface of the preform and the tool surface. This was used to determine the wrinkle amplitudes across the range of preforms. A global average thickness was determined for each preform, which was subtracted from the local thickness at each data point to establish the difference in height. This wrinkle amplitude is therefore independent of the number of plies in the preform, enabling direct comparison between preforms of varying thicknesses.

## Results

### Multi-ply Non-linear Bending Behaviour

**Fig. 7:**
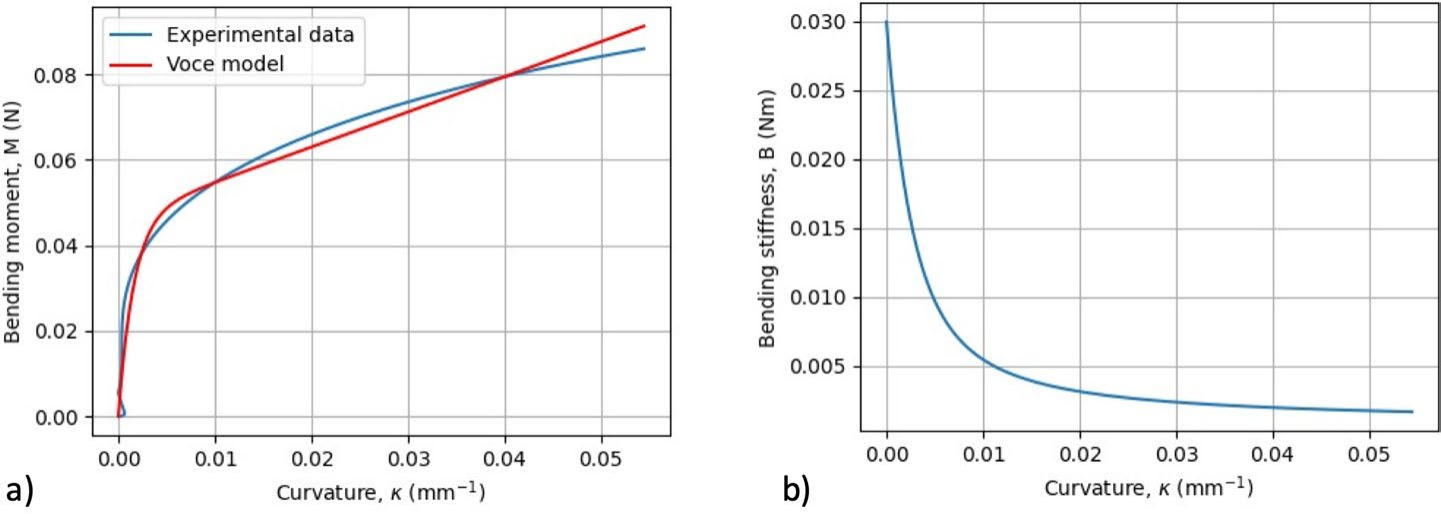
**a** Bending moment per unit width versus curvature for a single ply of FCIM359. **b** Bending stiffness per unit width versus curvature for a single ply of FCIM359

The bending stiffness of a single ply of FCIM359 is described by the bending moment per unit width versus curvature relationship shown in Fig. 7a. The mean root mean square error (RMSE) between the fitted curve and the experimental data was found to be 4.2% up to a curvature of 0.12 mm^− 1^. These results agree with previous bending data for the same fabric [26]. The unstable behaviour observed in the low curvature regions of the bilinear bending moment aligns with data reported for textile materials in the literature [27]. Figure 7b describes the non-linear bending behaviour of a single ply of FCIM359, where the bending stiffness decreases as curvature increases. The initial bending stiffness at 0 mm^− 1^ curvature was found to be 0.0076 Nm.

**Fig. 8:**
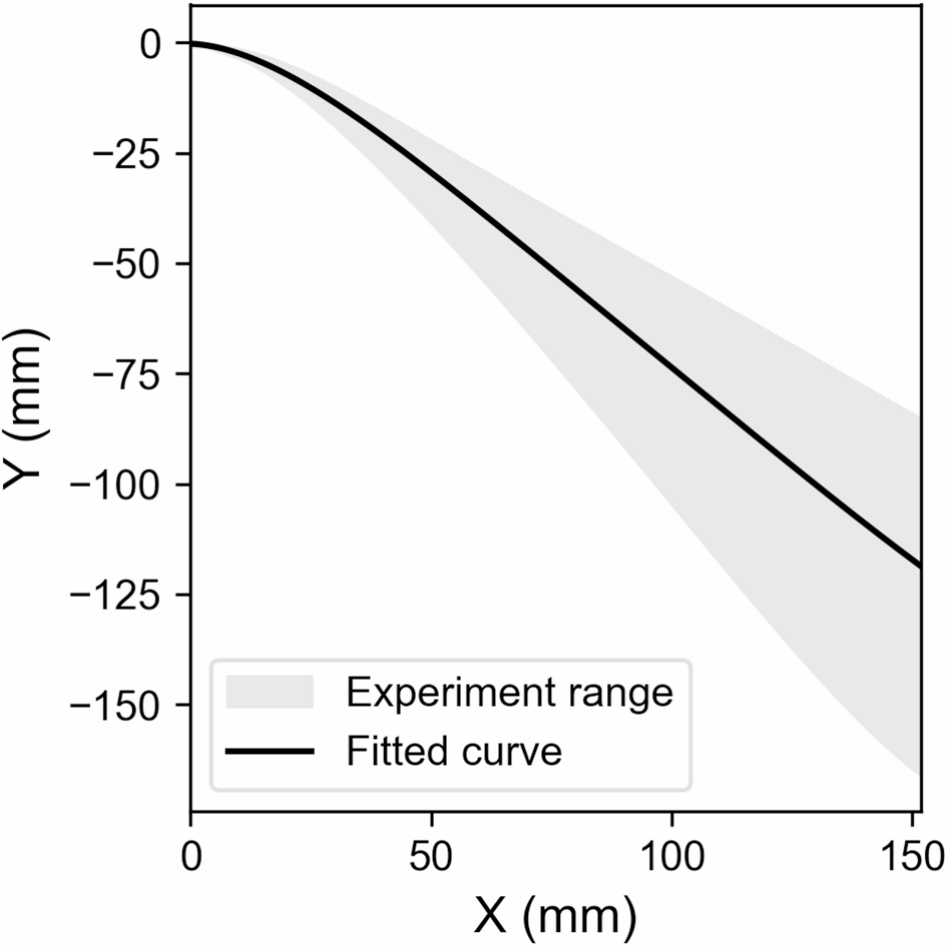
Free body diagram of the fitted curve from the 3D scan data for 16 [0°/90°] plies. Grey shaded region indicates the experimental range over 7 repeats

**Fig. 9:**
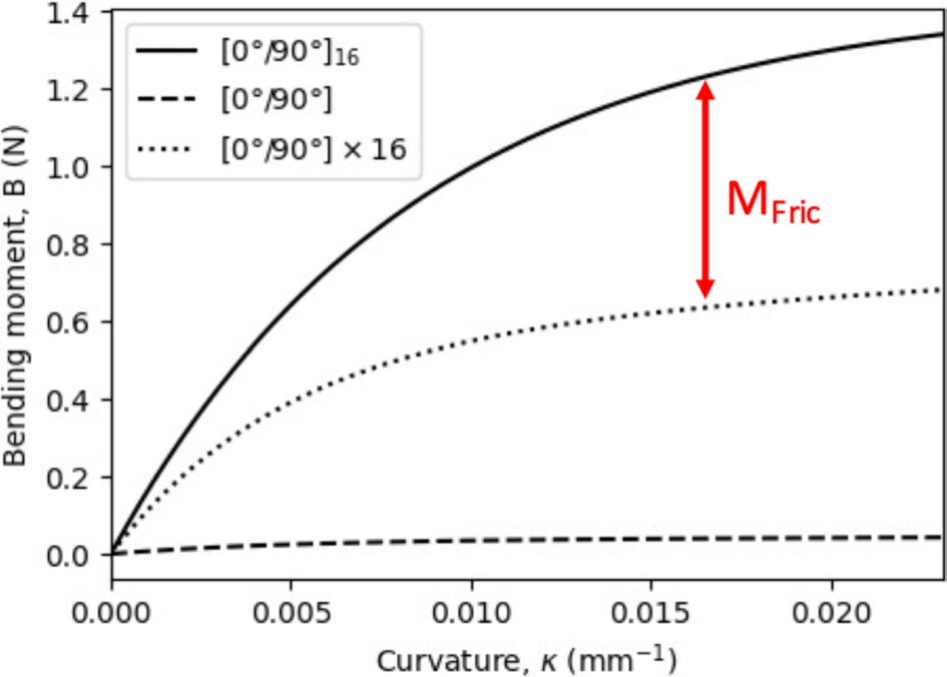
Curvature-bending moment per unit width curves for a 16-ply sample (solid line), a 1-ply sample (dashed line) and the summation of the bending moment for 16 individual plies of FCIM359 (dotted line). The difference between the bending result for the experimental 16-ply stack and the 16-ply theoretical summation is indicated in red as the frictional moment. All fibres are orientated at 0/90 degrees relative to the bending direction

The multi-ply stack exhibited non-linear bending behaviour similar to the curves produced for the single-ply, as shown in Fig. 8. However, for samples of the same length, the multi-ply samples produced a smaller curvature range due to the resulting increased bending stiffness. The bending behaviour for a multiply stack is shown in Fig. 9, where the bending moment for a [0/90]_16_ sample (solid line) is greater than the summation of the bending moment for each individual ply in the stack (dotted line), at any given curvature. The $\:{R}_{0}$, $\:{R}_{inf}$ and $\:{\kappa\:}_{lim}$ bending parameters for each case, as defined by Eq. (7), can be found in Table 3. This difference is defined as the frictional moment ($\:{M}_{Fric}$) and has previously been observed in [30]. It is caused by the inter-ply friction in the laminate and is a significant component of the overall bending moment. This inter-ply sliding resistance effectively increases the overall second moment of area of the system and consequently increases the perceived bending stiffness [36]. The dashed line in Fig. 9 shows the moment-curvature relationship for a single ply, which represents the behaviour expected from the lower bound case described by Eq. (1).

**Table 3:** Bending parameters of tested FCIM359 laminates, as defined by Eq. (7)

Description	Single-ply	16 x single-ply	Multi-ply
Layup	[0°/90°]	16 x [0°/90°]	[0°/90°]_16_
$\:{\boldsymbol{R}}_{0\:}$	*\:0.2927*	*\:4.6908*	*0.00015818*
$\:{\boldsymbol{R}}_{\boldsymbol{i}\boldsymbol{n}\boldsymbol{f}\:}$	*\:0.0361*	*\:0.5772*	*\:1.4240*
$\:{\boldsymbol{\kappa\:}}_{\boldsymbol{l}\boldsymbol{i}\boldsymbol{m}}$	*\:0.0049*	*\:0.0049*	*\:0.0083*

**Fig. 10:**
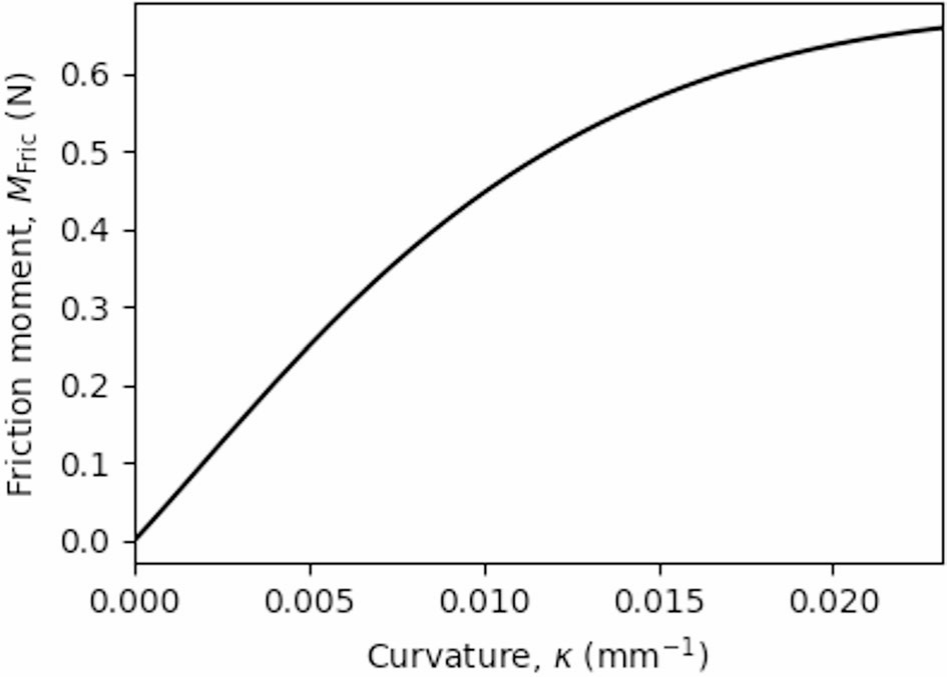
Curvature versus friction moment of a 16-ply [0/90] sample of FCIM359. The highest curvature occurs at the constrained end of the sample

Figure 10 shows the variation in the friction moment with curvature for the same 16-ply test (the difference between the solid and dotted black lines in Fig. 9), which also naturally follows a non-linear relationship. The highest values of the friction moment can be seen when the curvature is at its largest, which occurs near the constrained end of the sample. The frictional moment contributes a significant proportion of the total bending moment of a multi-ply stack, up to 51% at the maximum curvature value of 0.023 mm^− 1^. Therefore, any changes to the coefficient of friction at the interfaces between plies (such as those generated when normal load is applied) are expected to affect the bending stiffness of the ply stack.

### Multi-ply Shear Behaviour

**Fig. 11:**
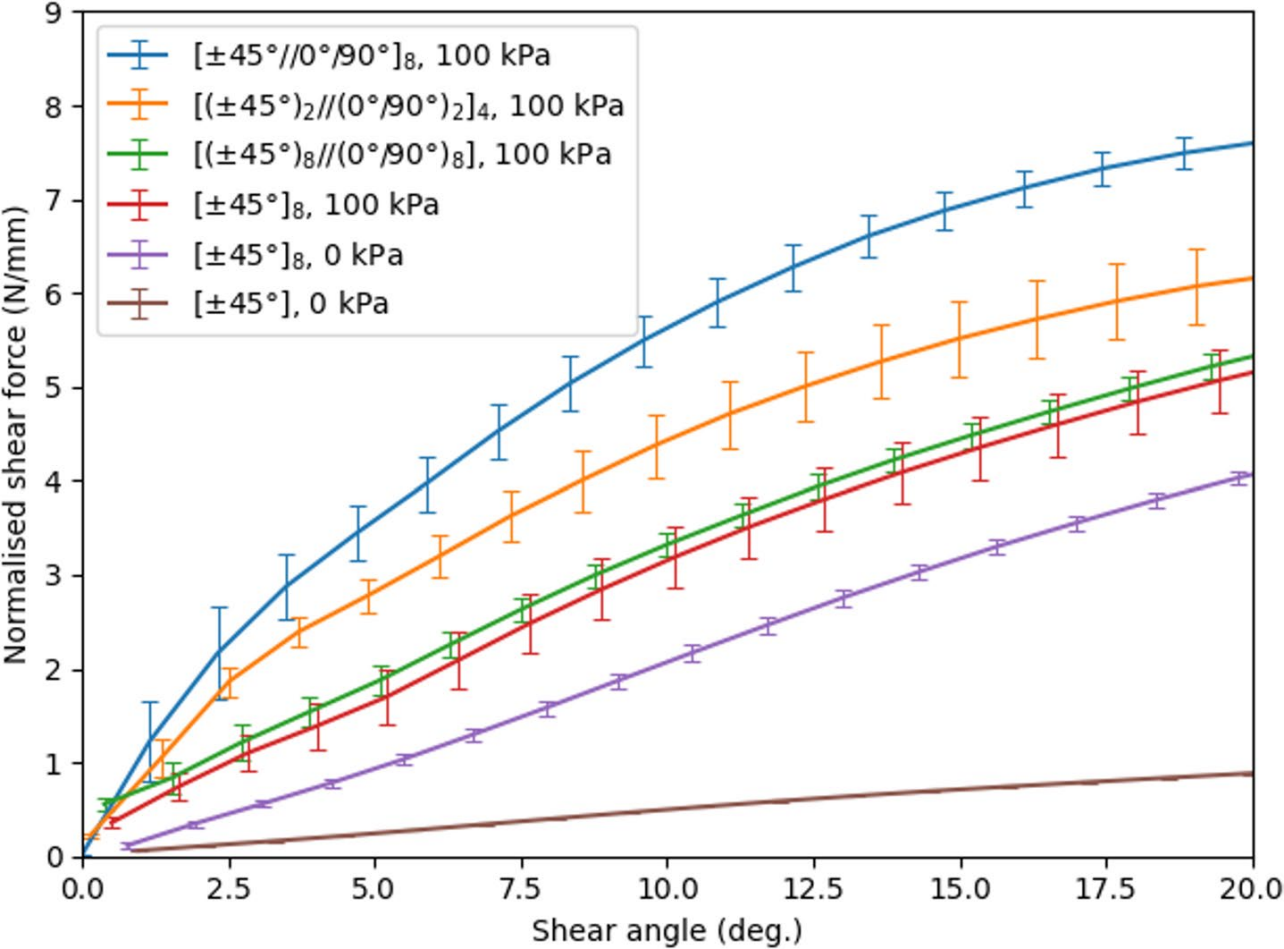
Normalised shear force versus shear angle for a range of multi-ply FCIM359 layups with two different compaction pressures (0 kPa and 100 kPa). Each curve represents the average response of 3 test coupons, where the error bars indicate ± one standard deviation

Figure 11 shows the shear force versus shear angle curves for a range of FCIM359 multi-ply layups, produced from the modified bias extension shear test. The results were normalised according to the method described by Cao et al. [37]. All curves represent the mean of at least three repeats and error bars indicate plus/minus one standard deviation.

The multi-ply layup exhibiting the lowest shear resistance was found to be a [± 45°]_8_ stack (purple curve), which was subjected to atmospheric pressure only (0 kPa). The lack of compaction (normal load) limits inter-ply surface interactions, avoiding any significant resistance to the shear behaviour of the fabric. The shear resistance of the 8-ply stack (red line) is consistently higher when vacuum compaction pressure is introduced. At an arbitrary shear angle of 20°, the shear force is 26% larger for the specimen held under vacuum compared to the specimen at atmospheric pressure. This increase is caused by greater intra-ply friction between the yarns at the shear rotation points, since every ply is at the same orientation and they are shearing in the same manner. The amount of relative inter-ply slip between the eight plies is therefore negligible.

The introduction of 8 additional [0°/90°] plies (indicated by the blue, orange and green lines in Fig. 11) increases the magnitude of the inter-ply frictional force in each case, which is dependent on the number of slip interfaces between plies of different orientations. It is assumed that the [0°/90°] plies do not shear since they are not clamped at the edges. Friction at the [0°/90°] and [± 45°] interfaces inhibit the shear deformation of the clamped [± 45°] plies, increasing the overall shear resistance of the laminate. According to Lawrence et al. [17], the dynamic inter-ply coefficient of friction varies between 0.5 (perpendicular fibre orientations) and 0.72 (parallel fibre orientations) for FCIM359 NCF when vacuum consolidation pressure is applied. This range drops to 0.35 to 0.41 when the vacuum pressure is reduced by 50%, indicating the pressure sensitivity of the inter-ply friction.

A “blocked” layup of [(± 45°)_8,_ [(0°/90°)_8_]] only has a single inter-ply interface where the ply orientations are dissimilar. The small increase in shear force for any given shear angle (3% greater at a shear angle of 20°) is greater than the mean coefficient of variation of either sample, indicating the increase in shear force is due to the introduction of the [0°/90°] material. Increasing the number of inter-ply slip interfaces to 7 magnifies this increase in shear resistance, producing an increase in shear force of 15% at a shear angle of 20°. Finally, an “interleaved” layup of [(0°/90°), (± 45°)]_8_ with 15 dissimilar ply-to-ply orientation interfaces results in a 42% increase in shear force at a shear angle of 20° compared to the [± 45°]_8_ layup. The magnitude of the shear force (at a shear angle of 20°) is proportional to the number of slip interfaces, closely following a quadratic relationship (R^2^ = 0.9986), as the sliding contact area increases with increasing slip interfaces.

### Double Diaphragm Forming Case Study

To assess the influence of friction on in-plane shear and out-of-plane bending behaviours during the forming of a multi-ply preform, a DDF case study was undertaken using the C-spar geometry described in Fig. 5. A 16-ply laminate was used to investigate the effect of increasing the magnitude of the frictional forces within the fabric stack. This was achieved by expanding the range of stacking sequences studied in Sect. 3.2, using 8 plies at [± 45°] and 8 plies at [0°/90°] relative to the longitudinal axis of the spar. These layups are described in Table 4. Each stacking sequence generates a different number of inter-ply slip interfaces (SI) to generate different levels of relative motion between the plies. It is possible to generate laminates with five different numbers of slip interface (1SI, 2SI, 3SI, 7SI and 15SI) using a 16-ply laminate with two different ply orientations.

**Table 4:** Laminate designs used to generate different frictional forces. Number of slip interfaces is indicated by the column heading and the position of the slip interface is indicated by the horizontal lines between rows. Ply #1 is closest to the tool surface. Orientations are shown in degrees from the principal axis of the C-spar geometry

	Number of Slip Interfaces (SI)						
Ply #	0	0	1	2	3	7	15
1	± 45	0/90	± 45	± 45	± 45	± 45	± 45
2	± 45	0/90	± 45	± 45	± 45	± 45	0/90
3	± 45	0/90	± 45	± 45	± 45	0/90	± 45
4	± 45	0/90	± 45	± 45	± 45	0/90	0/90
5	± 45	0/90	± 45	0/90	0/90	± 45	± 45
6	± 45	0/90	± 45	0/90	0/90	± 45	0/90
7	± 45	0/90	± 45	0/90	0/90	0/90	± 45
8	± 45	0/90	± 45	0/90	0/90	0/90	0/90
9	± 45	0/90	0/90	0/90	± 45	± 45	± 45
10	± 45	0/90	0/90	0/90	± 45	± 45	0/90
11	± 45	0/90	0/90	0/90	± 45	0/90	± 45
12	± 45	0/90	0/90	0/90	± 45	0/90	0/90
13	± 45	0/90	0/90	± 45	0/90	± 45	± 45
14	± 45	0/90	0/90	± 45	0/90	± 45	0/90
15	± 45	0/90	0/90	± 45	0/90	0/90	± 45
16	± 45	0/90	0/90	± 45	0/90	0/90	0/90

Two baseline laminates were formed using stacks of [0°/90°]_16_ and [± 45°]_16_ plies, containing no slip interfaces. Both laminates were successfully formed with no wrinkles (images not shown). The dominant forming mechanism for the [± 45°]_16_ was in-plane shear, as the primary yarns are required to rotate to conform to the areas of double curvature where the two ramped regions meet. Conversely, the dominant forming mechanisms for the [0°/90°]_16_ plies are out-of-plane bending and sliding, as continuous fibres run the length of the spar, which are required to draw into the ramped section, as indicated in Fig. 5. This was demonstrated through numerical modelling for the same geometry by Yu et al. [22], which showed negligible shear strain in the ramp region for the [0°/90°] plies. Despite the differences in forming mechanisms, both baseline laminates conformed fully to the tool without bridging or wrinkling defects.

**Fig. 12:**
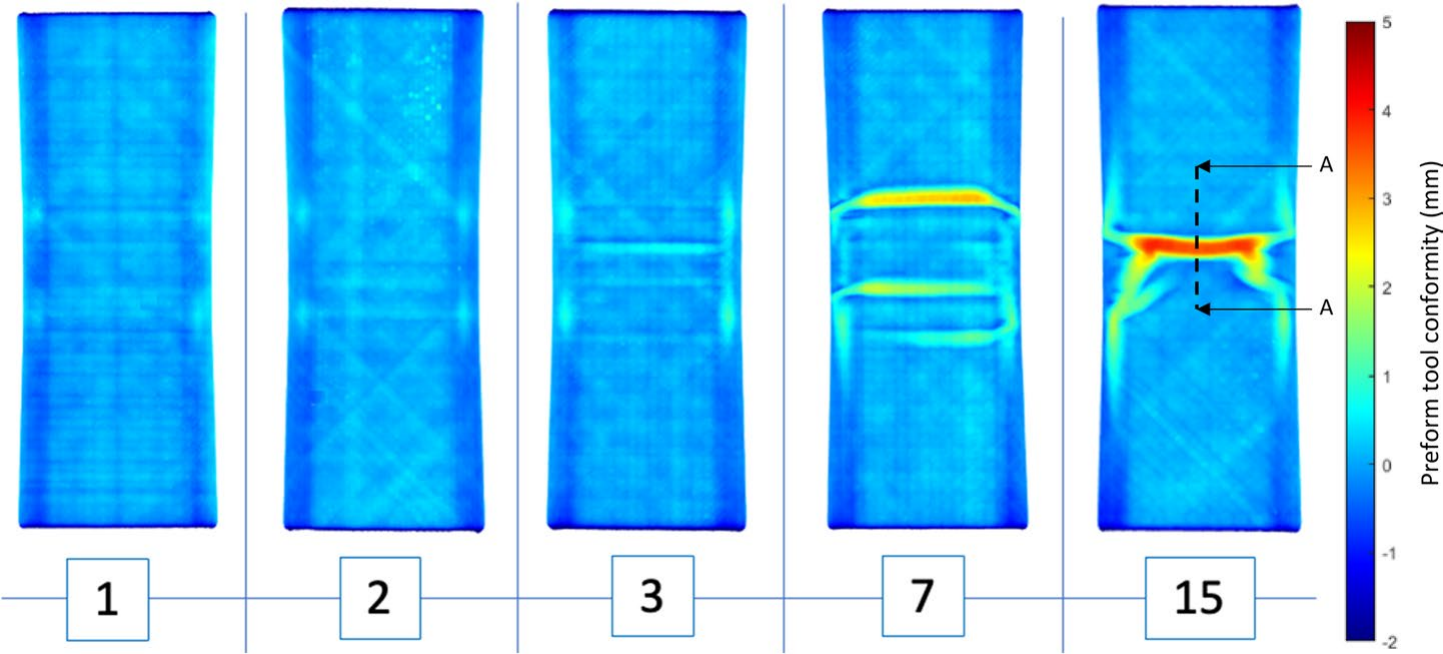
Comparison of wrinkle patterns for 16-ply preforms with an increasing number of slip interfaces. Number of slip interfaces indicated by number beneath each image. Contour indicates the distance between the outer surface of the preform and the expected defect-free preform shape containing 16 plies

**Fig. 13:**
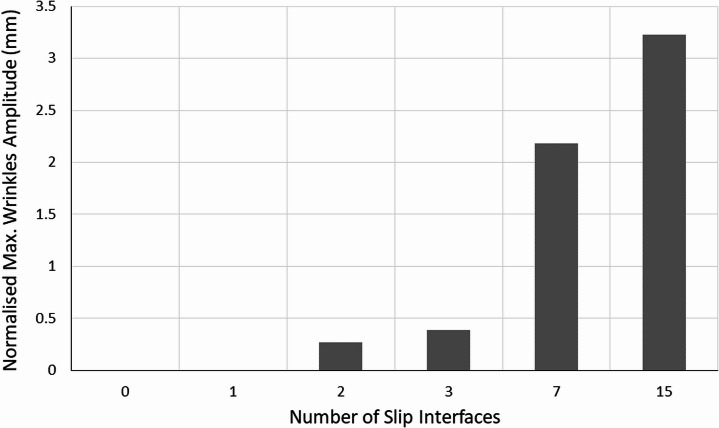
Normalised maximum wrinkle amplitudes for experimental DDF spar preforms. Maximum wrinkle amplitudes are normalised to the 1SI case

**Fig. 14:**
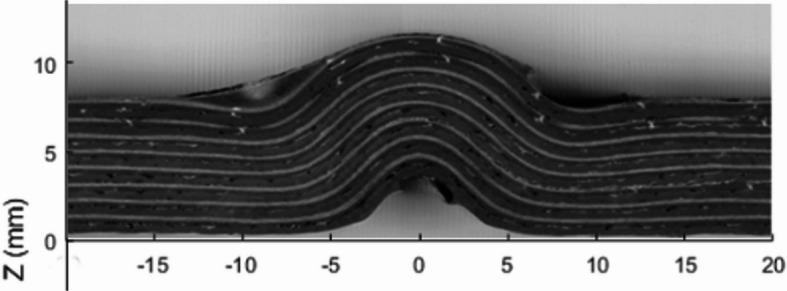
Cross section of the principal transverse wrinkle in the 15SI DDF case. Taken through Section A-A in Fig. 12. (All dimensions in mm)

These baselines were used to evaluate the severity of wrinkles in laminates containing an increasing number of slip interfaces. Figure 12 compares the full-field wrinkle patterns on the upper face of the C-spar with the expected surface profile from the defect-free laminates. Wrinkle amplitudes scale monotonically with the number of dissimilar orientation interfaces, which is consistent with the friction-limited shear across the stack. A comparison of the maximum wrinkle amplitudes is summarised in Fig. 13. The 1SI case shows good formability, producing no significant wrinkling. Wrinkles begin to form for the 2SI case, with a maximum wrinkle amplitude of 0.27 mm. The wrinkle amplitude continues to increase with increasing number of slip interfaces, with a maximum value of 3.3 mm for the 15SI case. A cross-section is shown in Fig. 14 through the largest transverse wrinkle for the 15SI case, confirming that the wrinkle exists in every ply, increasing in wavelength through the Z direction towards the outer surface of the spar.

The increase in wrinkle amplitude with increasing slip interfaces can be attributed to the increase in the total frictional force within the preform stack. The number of opposing deformation modes (inter-ply sliding, in-plane shear) at the interfaces between dissimilar ply orientations increases, therefore the overall resistance to deformation increases, forcing the fabric to buckle out-of-plane. According to Fig. 9, the [0°/90°]_16_ baseline laminate exhibits the highest bending stiffness because continuous fibres are running the length of the spar for all 16 plies, yet it remains fully formable without wrinkles. The increase in bending stiffness caused by the frictional bending moment (the combined effect of increasing the number of plies and increasing the applied compaction force from the vacuum) does not affect the formability of the [0°/90°]_16_ laminate. Therefore, it can be concluded that the increased resistance to shear deformation caused by the frictional forces at the slip interfaces is the principal mechanism responsible for the wrinkles in the other multi-ply layups in this study. The in-plane shear in both the [0°/90°] and [± 45°] plies is significantly constrained in the 15SI case compared with the 1SI case. Consequently, the fabric is forced to buckle out-of-plane to conform to the tool geometry.

It can be concluded that the level of out-of-plane wrinkling is directly related to the level of inter-ply friction, as this governs the degree of sliding that can occur at the slip interfaces when dissimilar ply orientations are used. Previous work has shown that the coefficient of friction is influenced by the fabric architecture, the relative fibre angle between adjacent plies [22] and the stitch pattern [17]. Factors leading to increased inter-ply friction are likely to lead to increased out of plane wrinkling as the number of slip interfaces increase.

## Conclusions

The mechanisms responsible for wrinkling defects during the preforming of thick multi‑ply NCF laminates were investigated using a series of novel characterisation tests alongside a double diaphragm forming (DDF) case study. The non‑linear bending behaviour of multi‑ply laminates was examined, showing that inter‑ply friction arising from fabric compaction and fibre nesting leads to a substantial increase in bending stiffness as laminate thickness increases.

Multi‑ply shear behaviour was evaluated under different levels of compaction pressure to replicate conditions experienced during DDF. Applying vacuum pressure (100 kPa) increased intra‑yarn friction, resulting in an average 18% rise in in‑plane shear resistance. The role of inter‑ply friction was further explored by interleaving additional fabric plies within the laminate stack. This demonstrated that shear resistance increases with the number of slip interfaces created between plies of differing orientations.

A multi‑ply DDF case study using a C‑spar tool geometry confirmed that increasing the number of slip interfaces in a 16‑ply stack significantly reduced laminate formability, leading to pronounced out‑of‑plane wrinkling defects. Based on shear distributions reported in previous simulation studies, this loss of formability was attributed primarily to increased in-plane shear resistance rather than elevated bending stiffness.

## Data Availability

The raw data required to reproduce these findings cannot be shared at this time as the data forms part of an ongoing study. The processed data required to reproduce these findings cannot be shared at this time as the data also forms part of an ongoing study.
